# Active Ageing in Europe: Adding Healthy Life to Years

**DOI:** 10.3389/fmed.2018.00123

**Published:** 2018-04-30

**Authors:** Giuseppe Liotta, Helena Canhao, Fabian Cenko, Rita Cutini, Ercole Vellone, Maddalena Illario, Przemyslaw Kardas, Andrea Poscia, Rute Dinis Sousa, Leonardo Palombi, Maria Cristina Marazzi

**Affiliations:** ^1^Department of Biomedicine and Prevention, University of Rome “Tor Vergata”, Rome, Italy; ^2^CEDOC, EpiDoC Unit, NOVA Medical School, Nova University of Lisbon, Lisbon, Portugal; ^3^Catholic University “Our Lady of Good Counsel”, Tirana, Albania; ^4^Sociology, University for Foreigners “Dante Alighieri”, Reggio Calabria, Italy; ^5^UOD Health Innovation, Campania Region, Naples, Italy; ^6^Department of Family Medicine, Medical University of Lodz, Lodz, Poland; ^7^Institute of Hygiene, University of Sacred Heart, Rome, Italy; ^8^LUMSA University, Rome, Italy

**Keywords:** active and healthy ageing, Europe, healthy life years, blue print initiative, frailty

## Background

The European Union (EU) is a conglomerate of more than 500 million people, 19.2% (approximately 100 million) of whom are older adults (1). The ageing population is triggering dramatic demographic, epidemiological, and anthropological changes, highlighting the importance of active and healthy ageing (AHA). In Europe, the most common household type is single occupancy (33.4% of the total number of households) (2). This household type also recorded the highest increase from 2005 to 2015 (3). These findings highlight several questions from both an individual and public perspective. Who will take care of the current generation as we become older? What types of health and social organisations should we develop to preserve the quality of life of an ageing population and sustain our health care systems over the medium and long term? Supporting AHA is one answer to these questions: an AHA population is a resource that benefits all of society. Maintaining a healthy ageing population may also lower demands for health care services. In addition, in many cases, older adults in good health are able to support their fellow generation and represent a strength dedicated to the common well-being.

## Active Ageing and Healthy Life Years

Active ageing is a multidimensional concept affected by several factors, including physical functionality, lifestyle, urban environment, and social inclusion (4, 5). In 2015, the World Health Organisation defined active ageing as “…the process of optimizing opportunities for health, participation and security in order to enhance quality of life as people age” and healthy ageing as “…the process of developing and maintaining the functional ability that enables well-being in older age” (6). The World Health Report on ageing underlined the role of public health strategies in building and maintaining health in older adults.

An operational definition of healthy ageing is still being debated, however, and consensus has not yet been achieved. McLaughlin and colleagues, who analysed the impact of different definitions of healthy ageing, concluded that a functional definition of health, i.e., free from symptomatic diseases and disabilities, may be acceptable (7). The valuable pragmatic approach supported by these researchers, however, does not account for the social dimension of “active” ageing. This social dimension is considered to be crucial because of its impact on developing and maintaining health at all ages. For example, the role played by social isolation as a risk factor for negative events in the older adult population, such as death, is well known (8–11). A more comprehensive approach to assess the process of AHA at the population level, therefore, should take into account several domains, including health status, income security, capability, and environment (12). This concept is consistent with the approach recently proposed by WHO, that describes healthy ageing as the result of the interaction between the physical and mental capacity of an individual (the intrinsic capacity), and the context of each individual’s life (the environment) (13).

Several models have been proposed to measure AHA, however, consensus is needed to be able to implement specific actions (14, 15). The healthy life year (HLY) expectancy, which accounts for the interaction of psychophysical and socioeconomic factors during the individual’s life course, could be considered a marker of AHA (16). In this view, HLY expectancy could be used as a measure to guide the development of the AHA process at the population level (17).

Although life expectancy at birth has increased by approximately 3 years over the past decade, disability-free life expectancy (i.e., the HLY expectancy) has not increased over this same time period (18). This phenomenon is not consistent across European countries, since in 12 countries out of 28 the HLY expectancy at 65 years is decreased from 2010 to 2014 (19). Moreover, the gap between life expectancy and healthy life expectancy is increasing. HLY expectancy is determined primarily by progressive impairment in performing activities of daily life (ADL), a measure of functional decline that is associated with frailty (20, 21). Frailty is associated with loss of autonomy in performing ADLs as well as health-related problems, institutionalisation, and/or hospitalisation, with negative influences on quality of life. From a public health perspective, frailty is a multidimensional issue resulting from changes in physical and mental health and functional status as well as lack of social and economic resources. There is evidence that functional decline is associated with lower psychosocial status, namely social isolation, malnutrition, and comorbidity (22–26), which are all determinants of frailty.

A number of initiatives are ongoing at the EU level to increase HLY expectancy at the population level *via* management of frailty at the community level. For example, the European Innovation Partnership on AHA gathers hundreds of public and private stakeholders from different backgrounds (e.g., academic and regulatory bodies, private entrepreneurs, non-government organisations, civil society) to brainstorm and execute innovative solutions for AHA to be exchanged and implemented across different settings within Europe (27). As another example, the Joint Action on Frailty witnesses the commitment of the European governments to set up common instruments and approaches to manage frailty in the community (28).

## Active Ageing and Prevention in Europe

Prevention is the likely key to increasing HLY expectancy (29). For example, approximately 50% of the reduction in mortality due to cardiovascular disease is the result of prevention-based activities, such as a change in lifestyle or smoking cessation (30, 31). Unfortunately, prevention programmes that are targeted to older adults are limited and chaotic, and not implemented in the framework of programmes with measurable outcomes. The European project “Pro Health 65+,” which seeks to determine effective methods for promoting a healthy lifestyle among older individuals, experienced some difficulties in collecting information on prevention programmes targeted to the older population. In many European countries, such as Italy, the public administration provides only a generic regulatory framework for promoting and facilitating such programmes (32). Even in countries with a national plan for the elderly, gathering data about either the implementation of different programmes or their impact is difficult (33), suggesting that more effort is needed to determine the impact of prevention programmes on the quality of life and demand of care among the older adult population in Europe.

Some communities within Europe have already developed successful programmes. In Barcelona, the Institut d’Investigació en Atenció Primària (IDIAP) Jordi Gol has developed a programme working with community nurses who provides elderly community members with advice on physical activities, diet, and medication schedule and also hold memory workshops: this programme reduced the use of health care services as well as the mortality rate of individuals over 65 years old in this community (34). The University Federico II in Naples has developed a programme that provides both information and communication technology (ICT) literacy and suggestions on diet and physical activities to the elderly community with positive outcomes among the over-65 population (35). In Rome, the Community of Sant’Egidio, a catholic NGO, has been running a programme called “Long Live the Elderly!” for the past 14 years. This programme, which applies social interventions to counteract social isolation in the over-75 population, was associated with a reduction in the use of health care services, health care costs, and mortality rate among this population (36). In addition, a study from the Campania Region of Italy has shown an improvement in bone health among older individuals who adhered to a Mediterranean diet (37). Thus, interventions aimed at lifestyle changes or counteracting social isolation are associated with reduced risk of death and reduced use of hospital services among older populations in Europe.

## Occupational Perspective on Active Ageing

The European Commission is strongly committed to connecting digitalization to AHA. The “Blueprint” initiative, a framework for strategic cooperation between key stakeholders (e.g., business, trade unions, research, education and training institutions, public authorities), outlined how digital innovation enabled by a functioning Digital Single Market can transform demographic changes into opportunities for Europe’s economy and society (38, 39) providing also the increase of job opportunities. The Digital Single Market is a strategy of the European Commission adopted in May 2015, to ensure access to online activities for individuals and businesses under conditions of fair competition and consumer and data protection. The grey digital gap (i.e., the lower proclivity of older adults to use personal computers or to communicate *via* the Internet), however, is deep; only approximately 45% of the over-65 population across Europe use the Internet at least once per week (40), and in 19 out of 28 countries in the EU, less than 50% of older adult citizens use the Internet at least once per week. Low Internet usage is due in part to lower education levels of older European citizens in these countries. In Portugal, for example, 77.3% of individuals over 65 years old have 4 or less years of education, and 84.5% have never used computers, videogames, or tablets (41). The “Blueprint” initiative, which aims to implement prevention programmes among European older adults, will, therefore, take significant time (e.g., 5–10 years) to implement among elderly EU citizens. It is likely that parallel efforts, aimed at health promotion and education and targeted at robust and pre-frail individuals, will be needed to maximise the impact of the “Blueprint” initiative over the next several years.

The health care market has not declined over the past 10 years despite the presence of a general economic crisis. The need for care has continued to increase as the population ages, and households are likely to invest more of their resources into this sector over the coming years. There is a window of opportunity, therefore, to implement programmes that proactively target the population at highest risk of frailty and functional decline, namely those over 75 years old. A large investment in health promotion and education that centres on strengthening health, ICT literacy, and social integration not only would serve as insurance for healthier old age and support for the sustainability of the European Health Systems, but also would offer significant occupational benefits to those who are interested in care services. Such programmes would require reorganisation of current models of care at the community level, and would also require individuals to change their view of AHA to that of a long-life approach based on health promotion and education.

## Conclusion

This debate emphasizes the key role of the determinants of health in AHA. In nineteenth century Europe, an improvement in socioeconomic conditions was the starting point for the most revolutionary demographic and epidemiologic transition in the contemporary age. Environmental sanitation and improved nutrition were the most important factors leading to this revolution at the population level. During this time, life expectancy increased from an average of 45 years to an average of approximately 80 years in most European countries. Europe was the first geographical area in the world to experience this success in life expectancy. Today, Europe continues to experience more advantages and more challenges with its ageing population than any other region in the world. Strengthening the social dimension of prevention programmes and changing the life styles represent key elements in improving quality of life and reducing the risk of loss of physical autonomy, especially among the over-75 population. These programmes can also provide occupational advantages across many European countries.

A strong effort is needed to improve the integration of the growing number of older adults to avoid the negative consequences of social isolation, malnutrition, and reduced physical activity. In the era of technological development and personalised medicine, environmental and nutritional factors together with healthy lifestyles represent the most powerful factors that determine the health of a population, which is encouraging because these are factors that can be influenced by health promotion and educational intervention. The current weakness of the public health approach to the promotion of AHA which threatens the quality of life of European older adults as well as the medium- and long-term sustainability of the health systems. However, Europe is well-poised to play a leading role worldwide in facing the challenges of increasing HLY expectancy by taking advantage of its assets in knowledge and innovation.

## Author Contributions

GL wrote the text. HC, MI, AP, and PK revised the text. FC, RC, EV, LP, MM, and RS contributed to the text with suggestions, comments, and proposals.

## Conflict of Interest Statement

The authors declare that the research was conducted in the absence of any commercial or financial relationships that could be construed as a potential conflict of interest. The reviewer AZ and handling Editor declared their shared affiliation.
